# Influenza A Virus in Swine: Epidemiology, Challenges and Vaccination Strategies

**DOI:** 10.3389/fvets.2020.00647

**Published:** 2020-09-22

**Authors:** José Carlos Mancera Gracia, Douglas S. Pearce, Aleksandar Masic, Monica Balasch

**Affiliations:** ^1^Zoetis Belgium S.A., Zaventem, Belgium; ^2^Zoetis Inc., Veterinary Medicine Research and Development, Kalamazoo, MI, United States; ^3^Zoetis Manufacturing & Research Spain S.L. Ctra., Girona, Spain

**Keywords:** influenza A, swine, epidemology, vaccines, role of pig

## Abstract

Influenza A viruses cause acute respiratory infections in swine that result in significant economic losses for global pig production. Currently, three different subtypes of influenza A viruses of swine (IAV-S) co-circulate worldwide: H1N1, H3N2, and H1N2. However, the origin, genetic background and antigenic properties of those IAV-S vary considerably from region to region. Pigs could also have a role in the adaptation of avian influenza A viruses to humans and other mammalian hosts, either as intermediate hosts in which avian influenza viruses may adapt to humans, or as a “mixing vessel” in which influenza viruses from various origins may reassort, generating novel progeny viruses capable of replicating and spreading among humans. These potential roles highlight the importance of controlling influenza A viruses in pigs. Vaccination is currently the main tool to control IAV-S. Vaccines containing whole inactivated virus (WIV) with adjuvant have been traditionally used to generate highly specific antibodies against hemagglutinin (HA), the main antigenic protein. WIV vaccines are safe and protect against antigenically identical or very similar strains in the absence of maternally derived antibodies (MDAs). Yet, their efficacy is reduced against heterologous strains, or in presence of MDAs. Moreover, vaccine-associated enhanced respiratory disease (VAERD) has been described in pigs vaccinated with WIV vaccines and challenged with heterologous strains in the US. This, together with the increasingly complex epidemiology of SIVs, illustrates the need to explore new vaccination technologies and strategies. Currently, there are two different non-inactivated vaccines commercialized for swine in the US: an RNA vector vaccine expressing the HA of a H3N2 cluster IV, and a bivalent modified live vaccine (MLV) containing H1N2 γ-clade and H3N2 cluster IV. In addition, recombinant-protein vaccines, DNA vector vaccines and alternative attenuation technologies are being explored, but none of these new technologies has yet reached the market. The aim of this article is to provide a thorough review of the current epidemiological scenario of IAV-S, the challenges faced in the control of IAV-S infection and the tools being explored to overcome those challenges.

## Nature of Influenza a Viruses

**Figure 1:**
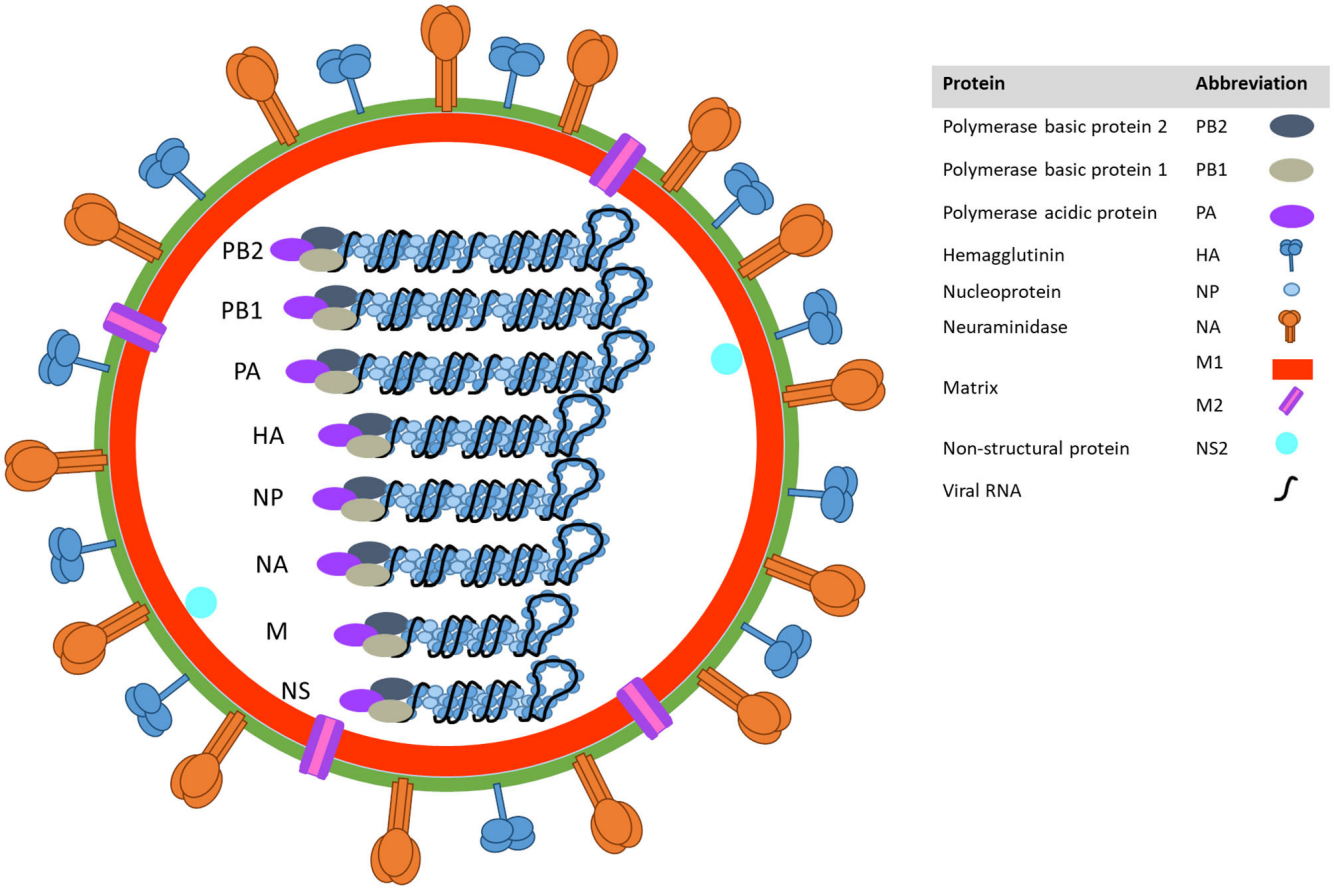
Influenza A virion structure.

Influenza A viruses belong to the family *Orthomyxoviridae* and their genome is composed of eight, negative-sense, single-stranded RNA segments (Figure 1). Two of those segments encode the two main surface proteins: the hemagglutinin (HA) and the neuraminidase (NA). These two viral proteins are major determinants of virus pathogenicity that play a crucial role in virus binding and release. In addition, HA and NA are used to classify the virus into subtypes according to their antigenic properties (1).

Because of their RNA viral genome, influenza viruses carry their own polymerase genes, which lack exonuclease proofreading capability. Therefore, influenza A viruses exist as dynamic populations with high mutation rates (2). Mutations that change amino acids in the antigenic sites of those proteins may allow influenza viruses to escape from pre-existing immunity. Such selective mutations produced in the antigenic domains of these surface proteins are responsible for a phenomenon known as “antigenic drift.”

Due to the presence of eight independent segments in the virus genome, simultaneous co-infection of a host cell with two or more different viruses can result in progeny viruses that contain novel combinations of gene segments from both parental viruses. This phenomenon is known as genetic “reassortment.” When genetic reassortment results in the emergence of a virus that contains a novel HA and/or NA protein, this is called “antigenic shift” (4). The combination of antigenic drift and shift poses a continuous threat to animal and human health increasing the challenge of developing efficacious vaccines (5).

## Influenza a Viruses in Swine

Influenza A viruses are an important cause of acute respiratory disease in pigs and contribute to Porcine Respiratory Disease Complex along with Porcine Reproductive and Respiratory Syndrome (PRRS), Porcine Circovirus Type 2 (PCV2), *Mycoplasma hyopneumoniae* and *Actinobacillus pleuropneumoniae*. Influenza A viruses of swine (IAV-S) target epithelial cells of the entire respiratory tract, replicating primarily in the lungs. As virus replication is restricted to the respiratory tract, virus transmission occurs only via the respiratory route. In pigs, influenza A infection lasts for 6–7 days and clinical signs such as fever, respiratory distress and weakness are resolved within a few days. Infection is usually mild and rarely causes death (1). However, this disease can cause a significant economic impact due to reproductive failure in sows due to the fever and weight loss in growing pigs.

Three different influenza A virus subtypes (H1N1, H3N2, and H1N2) are currently circulating in swine worldwide (6). However, the origins and the antigenic characteristics of these subtypes differ from region to region throughout the world.

**Figure 2:**
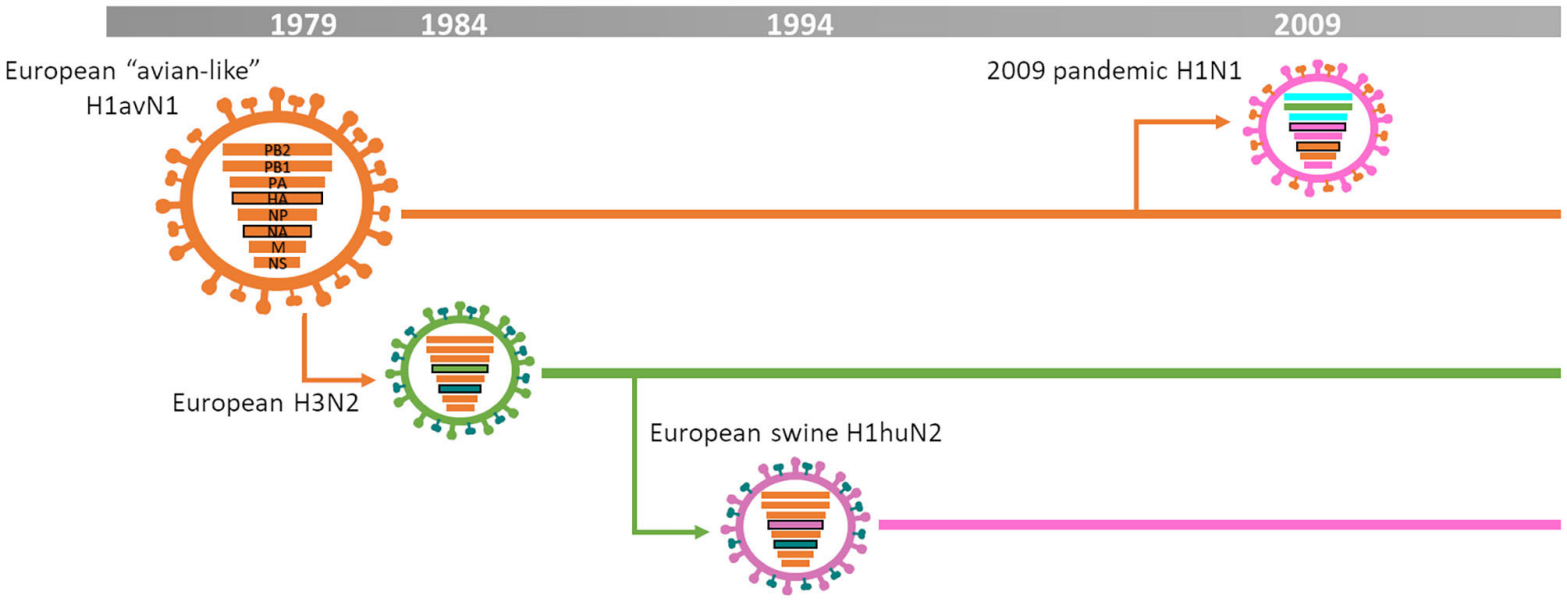
IAV-S epidemiology in Europe [based on (3)].

Figure 2 summarizes the IAV-S epidemiology in Europe. Briefly, the first significant influenza A virus outbreaks occurred in 1979 when an avian H1N1 virus jumped from wild ducks to pigs in Germany and Belgium (7). This virus is referred as European “avian-like” H1N1 (H1avN1), 1C clade based on the 2016 HA nomenclature for H1 subtype (8). H1avN1 viruses rapidly spread and became the predominant subtype throughout Europe (9, 10). During the mid-1980s, H3N2 strains spread and became the second endemic virus subtype in Europe. Those were reassortant H3N2 viruses containing the HA and NA from a descendant of the human 1968 “Hong Kong pandemic” H3N2 and the remaining genes from H1avN1 (9). In the mid-1990s, those H3N2 viruses reassorted with a human-seasonal H1N1 virus HA generating the H1huN2 virus lineage (11, 12). These viruses also became established throughout Europe and are classified as clade 1B (8). For many years, those three lineages co-circulated in the different European countries keeping the epidemiological situation rather stable (9, 13). However, this situation dramatically changed with the emergence of the 2009 pandemic H1N1 virus (H1N1pdm09) (13). This virus was the result of reassortment between a North American “triple-reassortant” swine influenza virus and a European H1avN1 (14). After its introduction in Europe, this H1N1pdm09 became established and widely reassorted with pre-existing H1N1, H3N2, and H1N2 subtypes, further complicating swine influenza epidemiology (6, 10, 15–19). Moreover, the H1N1pdm09 internal gene cassette extensively reassorted with domestic viruses in the UK and became the dominant backbone there (13).

**Figure 3:**
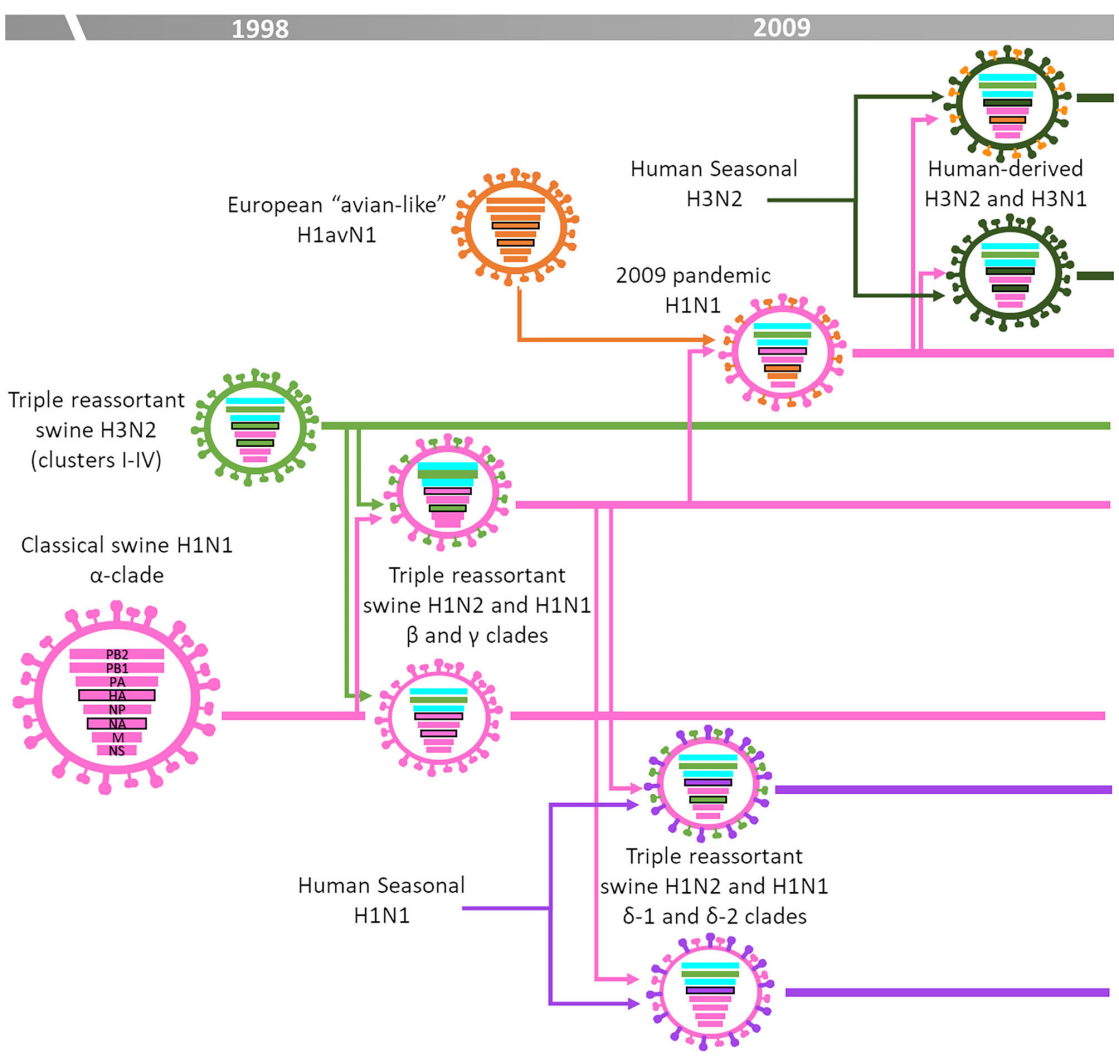
IAV-S epidemiology in the United States [based on (3)].

Figure 3 summarizes the IAV-S epidemiology in North America (Canada, Mexico, and the United States). In brief, in that region the epidemiological situation was stable until late 1990s. The “classical swine (cs)” H1N1, clade 1A (8), derived from the 1918 H1N1 pandemic (also known as “Spanish flu”), was the dominant subtype. Then, sometime in 1998, a novel H3N2 subtype emerged from the reassortment between csH1N1 virus genes (NP, M, and NS), human-seasonal H3N2 virus genes (PB1, HA and NA) and avian influenza virus genes (PB2 and PA) (20, 21). Due to the combination of swine, human and avian origin genes these viruses were designated “triple-reassortant” H3N2. This H3N2 subtype became established and further evolved into defined phylogenetic clades over time from Cluster-I to Cluster IV, which is the dominant cluster at the present day (22). The “triple-reassortant” H3N2 viruses further reassorted with csH1N1 leading to the generation and spread of novel “triple-reassortant” H1N1 or H1N2 viruses (23–25). These H1N1 and H1N2 lineage viruses related to the csH1N1 ancestor were designated as α, β, and γ clades (26). In addition, a minor clade (γ2-H1) was identified in 2013 and reported to be circulating in US herds since 1995 as a minor virus population (27). During the early 2000s, human-seasonal H1 and N2 genes were introduced into the US swine population by reassortment with the established “triple-reassortant” viruses. Those H1 viruses were antigenically different to those of the “classical swine” lineage and were classified as clades δ-1 and δ-2 (6). In 2009, the novel H1N1pdm09 emerged in Mexico. This was the first pandemic virus in the twenty-first century and was a reassortant containing M and NA genes derived from the European H1avN1 subtype and the remaining genes from a US “triple reassortant” H1 subtype (14, 28). The H1N1pdm09 efficiently spread in the human population but also spread in the North America swine population. Like Europe, the introduction and circulation of the H1N1pdm09 together with its reassortment with the endemic strains has deeply modified the scenario in North America (29, 30). During the 2010–2011 season, a novel human H3 virus lineage adapted to swine (31). This H3 was genetically and antigenically different from the cluster-IV lineage and currently coexists with them (32). In addition, H3N2, H1N1, and H1N2 viruses containing the M gene derived from the H1N1pdm09 spread throughout the US swine population and have been recurrently isolated from humans since 2011, raising public health concerns. These viruses were called “variant” viruses because of their ability to infect humans (29, 33).

In South America many different lineages likely circulated undetected for many years due to the lack of surveillance and reporting. Genetically and antigenically different lineages have been reported in the different countries. In Brazil, together with H1N1pdm09, various lineages containing human-seasonal H1N1 and H3N2 viruses surface genes and H1N1pdm09 internal genes were reported (34, 35). In Chile and Argentina multiple human-derived H1N1, H1N2, and H3N2 lineages have been reported, in addition to reassortants containing H1N1pdm09 internal genes (36–38).

Asia and the Pacific also show significant regional differences in swine influenza epidemiology. In China and Southeast Asia, which house more than 50% of the worldwide swine population, csH1N1 viruses were endemic until the 1990s. Later, due to pig imports from other continents, European H1avN1 and H3N2, and North American “triple reassortant” lineage viruses were introduced. The H1N1pdm09 also became endemic in the region after 2010. Those viruses widely spread and reassorted with endemic strains leading to a very complex collection of viruses (39, 40). In addition, other subtype viruses such as H3N8, H4N8, H5N1, H6N6, and H9N2 have been repeatedly reported in China. However, stable endemic status was never reached (41). In Australia, the introduction of specific lineages derived from human seasonal viruses such as H1N1 subtypes from 1977 and 1995 and H3N2 subtypes from 1968 and 2003 were detected in addition to H1N1pdm09. Three specific HA lineages derived from H3 (1995) and H1 (1977 and 1995) in combination with other human seasonal genes from the 1960s and the 2000s are the currently dominant subtypes (42, 43).

## Possible Role of the Pig in Pandemic Generation

According to the classical dogma, pigs may play a role in the adaptation of animal influenza viruses to humans. This hypothesis was first supported by Scholtissek and colleagues in 1985. They examined the rescue of temperature-sensitive (ts) nucleoprotein-gene mutants of an avian H7N1 virus by co-infecting chicken embryo fibroblasts with either avian, human or swine H3N2 isolates. They found that the ts mutants could be rescued by all avian viruses, by none of the human viruses and by two out of 10 swine viruses. In consequence, they proposed that the nucleoprotein of swine influenza viruses may have a broader host range when compared to human or avian viruses and that pigs were a potential “mixing vessel” for the generation of those reassortant viruses (44). This “mixing vessel” hypothesis was supported by two findings. First, subtype similarities between circulating human and swine influenza A viruses. Second, pigs could be simultaneously infected with avian, human and swine influenza viruses, which led to the generation and isolation of reassortants (45). In 1998, Ito and colleagues gave molecular support to this hypothesis by demonstrating the presence of the main sialic acid receptors for avian and human influenza viruses (Siaα2,3Gal and Siaα2,6Gal, respectively) in the pig trachea. Furthermore, they demonstrated that some “avian-like” swine influenza viruses acquired molecular traits of human adaptation by continuous replication in pig tracheal explants (46). This led to the hypothesis that the pig may also act as an intermediate host in which avian influenza viruses might gain mammalian adaptation traits. Still, later studies demonstrated that the presence of both sialic acid receptors in swine mimics that of human and ferrets (47–50). This sialic acid receptor distribution along with similar clinical manifestations and pathogenicity between swine and humans suggest that pigs could be an optimal model to evaluate influenza A virus infection and immunity with results that could have implications for human health (51, 52). More recent studies demonstrated that four serial passages of an avian H9N2 virus in pigs enhanced virus replication and transmission. However, efficient adaptation to reach endemic IAV-S replication and transmissibility parameters will likely need more adaptation (53).

In 2009, the emergence of the H1N1pdm09 virus from swine again caused concern that pigs act as a source of pandemics and stimulated additional research (14, 28, 54). Some groups suggested that also the previous pandemic viruses from twentieth century may have been generated by reassortment in a mammalian host, possibly swine (55, 56). An additional role for the pig in the generation of pandemic influenza viruses was then suggested. While the pig population may act as a reservoir for human-derived viruses circulating with lower drift rates, the human population is likely generating protective immunity only against recent seasonal strains with higher drift rates. Therefore, the human population is provided protection against recent seasonal strains but remains naive against old strains that are only circulating in swine (57, 58). A serological study demonstrated that infection immunity to recent human H3N2 viruses confers minimal cross-protection against European human-derived H3N2 viruses circulating in swine (59).

Today, the exact role of the pig in the interspecies transmission and the exact mechanisms under cross-species transmission remain unknown. Recent studies showed that human-to-swine transmission is key to understanding the evolution of influenza diversity in pigs and that more information exists on human to swine transmission than swine to human transmission (60). Therefore, this question should be taken as a One Health approach to avoid implicating swine as a source of human viruses.

## Strategies to Control Influenza a Virus in Swine

The most effective strategy to control and prevent IAV-S infection is vaccination. In contrast to other species such as humans or horses, there is no formal strain recommendation system for swine. This review will summarize approved and tested vaccine technologies for swine by dividing them in two main blocks: non-replicative vaccines, which are considered a safer approach as the lack of replication eliminates the risk of reassortment, and replicative vaccines, which can reassort with circulating field strains.

### Non-replicative Vaccines

#### Inactivated Vaccines

Inactivated vaccines are the traditional method to control IAV-S. Most current IAV-S vaccines contain whole inactivated viruses (WIV) with adjuvant for intramuscular injection and are either used in sows to protect them during gestation and their piglets during the suckling period or in growing pigs to decrease clinical disease (61). The goal of those vaccines is to induce serum neutralizing antibodies that target the viral HA (62). Antibodies are transferred to the mucosae of the respiratory tract to neutralize influenza viruses. Inactivated vaccines are locally produced, and they contain different strains in line with different antigenic and genetic virus strains circulating within each region.

In Europe, WIV vaccines are generally administered only to sows, yet only 10–20% of the sow population is vaccinated (61). As illustrated in Table 1, bi-valent vaccines containing H1avN1 and H3N2 subtypes were commercialized during the late 1980s. Nowadays, some of those vaccines are still commercialized in different European countries including Italy or Spain. Later, in 2010, a trivalent vaccine also containing H1huN2 was licensed, and this is still the main vaccine in most European countries. The most recent vaccine available in Europe was a monovalent H1N1pdm09 licensed in 2017.

**Table 1:** IAV-S vaccines commercialized in Europe from 1980s until 2020.

**Product name (manufacturer)**	**IAV-S strains**	**Type of adjuvant**	**Comments**
Gripovac (Merial^a^)	A/New Jersey/8/1976 (csH1N1) A/Port Chalmers/1/1973 (H3N2)	Oil	Production stopped
Suvaxyn Flu (Fort Dodge^b^)	A/swine/Netherlands/25/1980 (H1avN1) A/Port Chalmers/1/1973 (H3N2)	Oil	Production stopped
Respiporc Flu (IDT Biologika^c^)	A/swine/Belgium/230/1992 (H1avN1) A/swine/Belgium/220/1992 (H3N2)	Aluminum hydroxide-oil	Production stopped
Gripork (Hipra)	A/swine/Olot/1984 (H1avN1) A/Port Chalmers/1/1973 (H3N2)	Oil	Commercialized in Spain, Portugal, Ukraine, Greece, Russia, and Romania
Respiporc Flu 3 (IDT Biologika^c^)	A/swine/Hasselunne/2617/2003 (H1avN1) A/swine/Bakum/1769/2003 (H3N2) A/swine/Bakum/1832/2000 (H1huN2)	Carbomer	Commercialized in most European countries and the United Kingdom
Respiporc Flu pan (IDT Biologika^c^)	A/Jena/VI5258/2009 (H1N1pdm2009)	Carbomer	Commercialized in most European countries and the United Kingdom

a *Currently Boehringer Ingelheim*.

b *Currently Zoetis*.

c *Currently CEVA*.

Initial efficacy studies using inactivated vaccines in pigs were conducted using bi-valent formulations containing human-derived A/New Jersey/1976 (H1N1) and A/Port Chalmers/1973 (H3N2) strains. Interestingly, these vaccines were protective against non-related H1avN1 and more recent H3N2 IAV-S isolates. In fact, a Port Chalmers-based vaccine induced considerable antibody titers against H3N2 IAV-S strains isolated between 2008 and 2012 and significantly reduced clinical signs, replication in respiratory tissues and shedding after heterologous challenge with A/swine/Gent/172/2008 (H3N2) (63). Although A/New Jersey/1976-based-vaccine did not provide protection against A/swine/Gent/172/2007 (H1N1), other bivalent vaccines containing H1N1 isolates from the early 1980s and early 1990s showed a significant reduction of viral replication in the lungs. The most recent tri-valent vaccine did not show complete efficacy against the 2007 isolate despite containing a more recent H1N1 isolate (64). As expected and contrary to the tri-valent vaccine, none of the bi-valent vaccines conferred full protection against H1N2 (65). Finally, none of the commercial bi-valent or tri-valent vaccines were efficacious against H1N1pdm09. This gap was supposed to be filled by the commercialization of a monovalent vaccine containing H1N1pdm09. Nevertheless, a recent study demonstrated that this monovalent H1N1pdm09 vaccine does not confer full protection against antigenically distant H1N1pdm09 challenge (66). Studies investigating interference between European inactivated vaccines and pre-existing immunity are scarce. One serological study evaluated the antibody response induced in intranasally inoculated pigs (67). In this study, pigs were inoculated with one to three IAV-S belonging to the European endemic subtypes and later vaccinated with a commercial inactivated H1N1- and H3N2- based vaccine. Single vaccination of pigs previously infected resulted in a dramatic rise in hemagglutinating and neutralizing antibody titers to any of the viruses to which they were previously exposed. This suggests that a close antigenic relationship between vaccine and field strains is less important to provide heterologous protection in pigs previously infected with field strains. In addition, a more recent study demonstrated that heterologous prime and boost vaccination with European and North American (cluster IV) H3N2 subtype strains induced broadly cross-reactive antibodies that protected against homologous infection with both strains (68). The mechanisms behind that and whether those results can be extrapolated to the H1 subtype have yet to be elucidated.

In North America, vaccination against IAV-S is used more than in the EU with ~70% of the pig population being vaccinated (25). Table 2 summarizes the IAV-S inactivated vaccines commercialized in the US. The development and launch of inactivated vaccines in the US market coincided with the identification of novel circulating subtypes or clades. Thus, the first vaccine available was a monovalent vaccine developed against an α-H1N1 virus. Later, with the emergence of triple-reassortant H3N2 viruses, monovalent H3N2 and bivalent H1N1/H3N2 vaccines were released. Finally, due to the emergence of antigenically different H1 and H3 clusters, novel multivalent vaccines were launched. Also, in December 2009, a monovalent vaccine based on H1N1pdm09 was licensed (69). In addition to the commercial vaccines, around 50% of the inactivated vaccines used in the USA are autogenous, formulated to contain herd-specific strains.

**Table 2:** IAV-S inactivated vaccines commercialized in North America from 1994 until 2020.

**Product name (manufacturer)**	**IAV-S strains**	**Type of adjuvant**	**Comments**
MaxiVac FLU (Syntro Vet^a^)	α-H1N1	Oil	Production stopped
FluSure Legacy (Pfizer Animal Health^b^)	α-H1N1 Cluster I H3N2	Amphigen^®^	Production stopped in 2002
MaxiVac Excell 3.0 (Schering-Plow Animal Health^a^)	α-H1N1 β-H1N1 Cluster I H3N2	EMUNADE^®^	Production stopped
PneumoSTAR SIV (Novartis Animal Health)	α-H1N1 Cluster I H3N2	ImmunSTAR^®^	
FluSure XP (Pfizer Animal Health^b^)	A/swine/Iowa/110600/2000 (γ-H1N1) A/swine/Oklahoma/0726H/2008 (δ1-H1N2) A/swine/Missouri/069/2005 Cluster IV H3N2	Amphigen^®^	Formulation used in the United States 2008. Also in Canada, Mexico.
FluSure XP (Pfizer Animal Health^b^)	A/swine/Iowa/110600/2000 (γ-H1N1) A/swine/Oklahoma/0726H/2008 (δ1-H1N2) A/swine/North Carolina/031/2005 (δ2-H1N1) A/swine/Missouri/069/2005 Cluster IV H3N2	Amphigen^®^	Formulation used in the United States only (addition of δ2-H1N1 strain). Production stopped in 2016
FluSure XP (Zoetis)	γ-H1N1 δ1-H1N2 Cluster IVA H3N2 Cluster IVB H3N2	Amphigen^®^	Updated version of FluSureXP, commercialized from 2016, in US only
FluSure Pandemic (Zoetis)	A/California/04/2009 H1N1pdm09	Amphigen^®^	In US since 2009, final license in 2010
MaxiVac Excell 5.0 (Merck Animal Health)	β-H1N1 γ-H1N1 δ-H1N1 Cluster I H3N2 Cluster IV H3N2	EMUNADE^®^	

a *Currently Merck Animal Health*.

b *Currently Zoetis*.

In Latin America, IAV-S vaccines are primarily used in Argentina and Brazil. In Brazil the only vaccine commercialized is Flusure Pandemic, while in the remaining countries the same commercial vaccines as in the US are used.

Pigs enrolled in initial US-based efficacy trials were vaccinated twice with commercial monovalent csH1N1 vaccine and then challenged with a heterologous α-H1N1 (70, 71). Vaccinated pigs showed reduced clinical signs and lung lesions and nasal virus shed was either reduced or abolished. After the emergence of the H3N2 subtype, pigs vaccinated with commercial bivalent vaccines showed reduced clinical signs, pneumonia and viral excretion when challenged with a heterologous H1N1 (72). In contrast, although the same bivalent vaccines containing cluster I H3N2 IAV-s reduced clinical signs and lung lesions after challenge with a heterologous cluster III H3N2 virus, they failed to significantly reduce virus shedding (73). This lack of efficient protection was explained due to the genetic divergence between cluster I vaccine strain and cluster III challenge strain, which showed ~93% homology at the amino acid level (74). From early 2000's, both endemic H1 and H3 subtypes showed increased genetic and antigenic diversity which made controlling the disease with inactivated vaccines more challenging. For instance, pigs vaccinated with an experimental vaccine containing A/swine/Iowa/1930 (α-H1N1) strain were not fully protected against challenge with a heterologous A/swine/Minnesota/00194/2003 (γ-H1N2) strain (75). Moreover, in the heterologous challenged group, three out of nine pigs had significantly higher percentages of lung lesions when compared to the other groups. This phenomenon called vaccine-associated enhanced respiratory disease (VAERD) and was repeatedly reported with other H1N1 clade combinations, such as 2009H1N1pdm and δ1-H1N1, with both viruses used either as vaccination or challenge (76, 77). Later studies demonstrated that VAERD was related to the use of whole inactivated vaccines containing divergent HA and NA strains to those of the challenge viruses but also by the type of adjuvant used (78, 79). Interestingly, this phenomenon was never described in European vaccine studies. These results suggested that prediction of protection based on HA similarity was unreliable. Vaccination with the first version of the multivalent FluSure XP significantly reduced and delayed the level of β-H1N1 virus transmission virus from shedders to vaccinated animals compared to non-vaccinated animals but to a lesser extent than animals vaccinated with an homologous vaccine, which prevented this transmission completely (80). Another study performed with the same vaccine using A/swine/Illinois/02450/2008 (α-H1N1) as challenge showed partial protection demonstrated by significant reduction of virus present in bronchoalveolar lavages (BALF), nasal secretions and lungs, but no reduction in lung lesions (81). With the spread of 2009H1N1pdm in the US, three commercial vaccines were evaluated for their ability to induce protection. Although the 2009H1N1pdm HA belongs to γ-H1N1 clade and the three tested vaccines contained γ-H1N1 strains, none was able to confer complete protection and high levels of cross-reactive antibody titers (82). Challenge studies were also performed to evaluate the degree of heterologous protection against H3N2 provided by the multivalent vaccines. The conclusions achieved from those studies were that vaccines containing cluster IV H3N2 provided significantly better protection to circulating cluster IV H3N2 viruses when compared to older vaccines containing cluster I H3N2 strains (29, 83). Few studies demonstrated that the presence of maternally derived antibodies (MDAs) does not confer protection against heterologous challenge strains (84). To understand the different vaccine scenarios in the US and Europe, it is important to understand the different regulatory framework needed to approve new vaccines. In Europe, the European Medicine Agency (EMA), requires demonstration of vaccine efficacy through experimental vaccination-challenge studies (using heterologous challenge) against each vaccine subtype following the requirements of the European Pharmacopeia. In contrast, in the USA, the United States Department of Agriculture (USDA) allows the evaluation of the immunogenicity of additional or updated strains by serology only (69). This gives US manufacturers the opportunity to address vaccine updates in a more flexible manner when compared with their European counterparts.

Literature regarding availability of influenza vaccines in Asia is scarce and reports vary from country to country. In China, at least four inactivated adjuvanted licensed vaccines are available. Those vaccines are manufactured by local companies and are either H1N1 monovalent or H1N1, H3N2 bivalent products. Inactivated vaccines based on local strains are also mainly used in Japan and South Korea. In Japan, the main commercialized bivalent vaccine contains H1N1 and H3N2 strains isolated in the late 1960s and 1970s. In South Korea, there are three inactivated vaccines available, two of which are trivalent containing strains from 2004 to 2005. In both Asian countries, SIV vaccines contain mainly non-oil-based adjuvants.

#### Viral Vector Vaccines

In the late 2000s, the emergence of H1N1pdm09 both in pigs and humans and the isolation of variant H3N2 IAV-S from humans highlighted the need of a rapid response immunization strategy for pandemic influenza outbreaks. Alphavirus replicon particles containing IAV-S structural gene segments were included in that strategy because they allow for quick strain updates. Alphavirus replicon particles are propagation-defective, single-cycle vectors which deliver genetic material into the cytoplasm of the cell but cannot spread from cell to cell (85). The first recombinant product approved for IAV-S vaccination in the USA was an alphavirus-derived replicon particle vaccine licensed by Harrisvaccines (currently Merck Animal Health) in the early 2010s (“Swine Influenza Vaccine RNA,” Harrisvaccines, Inc. Ames, IA, USA) (86). This product consisted of an attenuated Venezuelan-equine encephalitis virus, which was replication-defective due to the substitution of structural genes by the HA of a North American cluster IV H3N2 IAV-S. This product was administered intramuscularly in a priming-boost schedule with a 2–3 weeks interval between each vaccination. After homologous challenge, vaccinated pigs showed reduced amount of viral RNA in nasal swabs and BALF, reduction of clinical signs, gross and histological lung damage. However, protection was not efficacious in the presence of MDAs (87). The homologous protection of this technology was confirmed by Vander Veen et al., which also demonstrated protection using the same platform expressing recombinant H1N1pdm09 HA (88). In the same study, a replicon-particle vaccine expressing a cluster IV H3N2 derived-NP gene was able to decrease nasal shedding and viral load in pigs after heterosubtypic challenge with H1N1pdm09. Later another study aimed to test the efficacy of a monovalent and bivalent combination of the vaccine expressing two different H3N2 HA genes against homologous and heterologous challenge (89). One of the monovalent vaccines provided good protection against homologous and heterologous challenge while the other monovalent vaccine conferred significant protection only against the homologous challenge. In contrast, pigs vaccinated with the bivalent vaccine showed minimal lung lesions and low or undetectable virus in lungs and nasal swabs after challenge. Another advantage of this vaccine platform is that it could be paired with diagnostic strategies of differentiating infected from vaccinated animals (DIVA).

An additional viral vector strategy explored in swine was the use of replication-defective human adenovirus serotype 5 (Ad5) as vector. This technology is based on the deletion of two segments of the Ad5 virus genome creating a replication defective phenotype and space to insert the desired extraneous genes. The HA and the NP genes of a cluster I H3N2 IAV-S were inserted into Ad5 and tested for vaccine efficacy in pigs (90). A single intramuscular dose of Ad5-HA alone or combined with Ad5-NP induced high levels of hemagglutination inhibition (HI) antibodies. Pigs vaccinated with the combination were completely protected against heterologous challenge as shown by lack of virus shedding and lung lesions. On the other hand, pigs vaccinated with Ad5-HA or Ad5-NP alone showed partial or no protection, respectively. This Ad5-HA + Ad5-NP combination could also be delivered with a needle-free device, but results were similar when compared to those of IM injection (91). The efficacy of the Ad5-HA + Ad5-NP combination in the presence of MDAs was also tested (92). A prime-boost vaccination strategy with the Ad5-HA + Ad5-NP combination followed by a commercial bivalent vaccine conferred protection against a heterologous H3N2 challenge in presence of H3N2-specific MDAs. In addition, a recombinant Ad5 encoding H1N1pdm09 HA gene was used to vaccinate pigs with a single intranasal (IN) dose (93). The vaccine induced mucosal antibodies and conferred solid protection against homologous challenge. However, immune response generated was only partially cross-protective against a heterologous challenge with a δ-H1N2 virus.

Other viral vectors have been experimentally tested in swine. Vaccination with recombinant equine herpes virus-1 or swinepox vectors expressing the HA genes of IAV-S protected against homologous challenge (94). However, those studies did not evaluate protection against a heterologous challenge or the impact of MDAs on vaccine performance. In a more recent study, pigs were vaccinated either with vesicular stomatitis virus- or with classical swine fever-derived replicon particles expressing the NP of a European H1N1 IAVs (95). Both vector vaccines elicited a potent antibody and T-cell response and were efficacious against homologous challenge. However, although antibodies and T-cells were cross-reactive, they did not provide protection against heterologous H1N2 infection.

#### Other Non-replicative Vaccine Technologies Tested in Swine

Exploration of DNA plasmid vaccines against influenza began in the 1990s as an alternative to avoid many issues associated with egg-based vaccine production, which was the main production method for inactivated influenza vaccines at the time (96). DNA vaccines consist of an antigen-encoding gene cloned into a non-replicative expression plasmid that is delivered into the host. This platform offers the advantage that several antigens can be combined in a single plasmid and that they are expected to generate cell-mediated and humoral immunity even in presence of MDAs. Several studies evaluated the immune response generated and protection conferred by DNA vaccines in pigs. DNA vaccines based on different gene combinations (mainly HA) demonstrated good degrees of protection against homologous challenge (70, 71, 97–99). Needle-free and IM delivery methods were tested to be successful, but recent studies evaluated needle-free delivery as it was claimed to be safer and easier to administer for large scale vaccination (97–99). The combination of priming with a DNA vaccine and boosting with an inactivated vaccine conferred significantly better protection than only two doses of DNA vaccine (71). However, heterologous cross protection was demonstrated even in presence of MDAs after two doses of DNA vaccine (99). The major handicap of those vaccines is that large doses of DNA and several vaccination doses were required to confer protection.

Another technology explored is the vaccination with HA trimers. In the context of the 2009 pandemic, another research group in the Netherlands evaluated the immune response and the protection generated against H1N1pdm09 in pigs after vaccination with recombinant H1N1pdm09 HA trimers (100). Upon double vaccination, pigs vaccinated with HA trimers were almost completely protected against challenge virus. Only low levels of virus replication were detected in the pig's respiratory tract. This finding was in line with the high levels of HI and virus neutralizing antibodies found against the homologous strain. Although heterologous challenge was not performed, HI cross-reactive antibody levels against H1avN1 and H1N2 were lower when compared to those raised against the homologous strain. Therefore, lower levels of protection may be expected.

The primary function of the influenza A virus M2 protein is to act as an ion channel for disassembly of the viral core, but also as a secondary conserved antigenic site in contrast to HA and NA antigenic sites, which are less conserved. Therefore, recombinant vaccines based on the M2 protein were proposed as universal influenza A vaccine candidates (101). This strategy showed promising results in mice (102, 103), but M2 based vaccines alone were not able to confer significant protection (104, 105).

### Replicative Vaccines

#### Live-Attenuated Virus Vaccines

Live-attenuated influenza virus (LAIV) vaccines consist of viruses produced by reverse genetics genetically modified to reduce viral replication. LAIV vaccines are administered directly to the respiratory mucosa by intranasal (IN) administration, which mimics natural infection and activates both mucosal and systemic immune responses. Mucosal antibodies, such as IgAs, are important to control IAV-S, and the cell mediated immune response induced by the replicating LAIVs is essential for broader cross-protection against natural infection as T cells mostly recognize conserved epitopes. Three different LAIV strategies have been tested in swine.

#### Attenuation by Non-Structural NS1 Protein Truncation

The goal of this technology is to hijack the ability of the virus to evade host cell type I interferon (IFN)-mediated antiviral response and to restrict virus replication. This is achieved by the deletion of 126 amino acids from non-structural NS1 protein, which is only expressed in virus-infected cells. This deletion was applied to a North American cluster I H3N2 (A/swine/Texas/4199-2/1998) strain that resulted in the absence of or minimal lung lesions and significantly lower virus titers in BALF when compared to the wild-type inoculated group. Interestingly, the attenuated virus had strong immunogenic properties in spite of its lower levels of replication (106). This immune response, generated after IN inoculation, was composed of high levels of mucosal IgAs and systemic cell mediated immune responses, as well as modest levels of systemic neutralizing antibodies (107–111). The LAIV vaccine conferred strong protection against homologous challenge in influenza-naïve pigs and nearly complete protection against the heterologous cluster II H3N2 (A/swine/Colorado/23619/1999), which is antigenically different (108). In contrast, after challenge with a heterosubtypic H1N1, vaccinated animals showed no reduction in lung lesions and a slight reduction of virus titers in BALF and nasal swabs at 5 days post challenge (107, 108). In addition, the NS1 LAIV vaccine showed partial protection in piglets with MDAs without inducing VAERD (112, 113). Since 2017, a NS1 LAIV vaccine became commercially available in the USA for use in pigs from 1 day of age. Ingelvac Provenza (Boehringer Ingelheim, St. Joseph, MO, USA), which is a bivalent product containing two reverse genetic generated LAIVs: one cluster I H3N2 virus based on A/swine/Texas/4199-2/1998 with the NS1 truncation and one virus containing the same attenuated internal gene cassette derived from the H3N2 strain but with the HA and NA derived from a γ2 beta-like H1N1 strain (A/swine/Minnesota/37866/1999). This vaccine was efficacious in reducing virus nasal shedding after challenge with heterologous strains, either H1N1 or H3N2, with and without presence of MDAs (113, 114). However, a recent phylogenetic study done in the US with samples collected in 2018 found reassortant strains containing LAIV vaccine strain genes in combination with US endemic field strain genes (115). These data indicate that viral reassortment is possible with LAIV vaccines. Further research will be required to evaluate its impact to the IAV-S epidemiology.

#### Attenuation by Polymerase Genes Mutations

Influenza virus polymerase complex is composed of polymerase basic 2 (PB2), polymerase basic 1 (PB1) and polymerase acidic (PA). Those three subunits working together are responsible for virus replication in the host cell (116). Previous studies in humans and horses identified that certain mutations in the viral polymerase PB1 and PB2 genes caused impaired polymerase activity and reduced replication at the temperature of the lower respiratory tract (117, 118). These cold-adapted and temperature sensitive (ts) mutations were also evaluated in a cluster I H3N2 IAV-S confirming the restricted virus growth in the respiratory epithelium of pigs (119, 120). Viruses were generated by reverse genetics to contain the ts internal gene cassette and several different HA and NA combinations. For instance, a single IN vaccination with the ts internal gene cassette combined with H1N1pdm2009 conferred sterilizing immunity against homologous challenge (120). Another study compared the efficacy in pigs of three US commercial vaccines each containing different H3N2 strains against two different LAIV vaccines, one with a NS1-truncated cluster I H3N2 strain and the other with a ts cluster IV H3N2 (83). After two doses all vaccines conferred significant protection against a heterologous cluster IV H3N2 challenge strain. However, only the ts-LAIV vaccine prevented aerosol transmission to indirect contact pigs. Another study compared the effects of heterologous challenge with a δ2-H1N2 strain after vaccination with two different H1N1pdm09 vaccines, a recombinant HA subunit vaccine or a ts-LAIV (121). The ts-LAIV partially protected pigs, as demonstrated by reduced virus shedding and faster viral clearance. In contrast, pigs vaccinated with the subunit vaccine developed more severe lung lesions right after challenge, which was consistent with VAERD. This absence of VAERD in pigs vaccinated with ts-LAIVs was further confirmed by another study (122). Although there are no specific studies that evaluate the efficacy of ts-LAIV vaccines in the presence of MDAs, the nature of the immune response generated by LAIV vaccines, which is mainly composed by mucosal antibodies and a cell-mediated component, suggests low levels of interference compared to the interference observed against inactivated vaccines. Additionally, there are no reports available that evaluate the potential of this vaccine to reassort with endemic field strains.

#### Other Technologies of Attenuation

Influenza A virus HA protein is synthesized as a precursor (HA0), which in order to become infectious must be cleaved by host proteases, usually trypsin, into HA1 and HA2. In IAV-S this process is usually mediated by trypsin-like proteins and it is essential for the virus to efficiently bind and replicate in the host cells (123). The modification of the HA cleavage site to be activated by elastase enzyme instead of trypsin resulted in virus attenuation due to the scarce presence of elastase in the host tissues when compare to the trypsin (124). Elastase-dependent mutants viruses based on a Canadian avian-like H1N1 strain (A/swine/Saskatchewan/18789/2002) (125), did not induce clinical signs or virus shedding in inoculated pigs. Those elastase-dependent strains generated robust cell-mediated and mucosal antibody responses after two IN or IT doses (126). After challenge, those H1N1 based LAIVs conferred robust protection against homologous and heterologous challenge strains (126, 127). However, only partial protection was described when challenged with an heterosubtypic H3N2 subtype (126). The same group generated one novel virus containing two HAs, an H1 and H3 in the genetic context of the previous H1N1 LAIV (128). This mutant was generated by fusing the H3 HA ectodomain of a triple reassortant H3N2 to the N1 NA transmembrane and cytoplasmic tail of the A/swine/Saskatchewan/18789/2002 to replace NA ectodomain and ultimately attenuate the virus. Like the previously described elastase-sensitive construct, the new chimeric H1–H3 vaccine candidate was highly dependent on presence of exogenous neuraminidase to subsides lack of NA viral function. The rationale behind this construct was to increase the level of cross-protection against heterologous H3N2 viruses with a single bivalent vaccine construct. After two vaccinations the novel LAIV induced antigen-specific systemic and mucosal antibody responses in the respiratory tract. In addition, vaccinated pigs had no or minimal lung lesions and undetectable levels of virus in the lungs after challenge either with H1N1 or H3N2 IAV-S strains. However, those results should be carefully interpreted as the H3N2 challenge virus was also undetected in the lungs of 4 out of 5 pigs from the challenge control group. Those vaccines proved to protect in presence of MDAs and VAERD was never reported (129).

## Conclusions

IAV-S can cause important health issues in pigs and the subsequent economic damage to the swine industry. Although only three subtypes of IAV-S are circulating the origins, genetic and antigenic diversity of those viruses show great regional differences. IAV-S populations are highly dynamic. However, the impact of IAV-S may not be exclusively related to swine industry. The first pandemic virus from the twenty-first century was caused by an influenza A virus generated in swine containing genes from avian, human and swine origins, and “variant” viruses have been repeatedly isolated from humans since 2010 (14, 33). Pigs and humans share the same influenza receptors pattern in their respiratory tract (49) and inter-species transmission of influenza A viruses from pigs-to-humans and from humans-to-pigs occur in both directions (60). Therefore, efficient prevention and control of IAV-S may not be only a benefit for swine health but for human health.

Currently, the main tool to control IAV-S infection is by vaccination. The desirable vaccine should be easy and safe to administer, generate a robust immune response to confer heterologous or even heterosubtypic broad protection, function in the presence of MDAs or active immunity, and not induce VAERD. None of the vaccines described here comply with all the characteristics described. The inoculation route has an impact in the immune response generated. For instance, IM or intra-dermal route vaccines generate higher humoral responses based in HA neutralizing antibodies, which are very strain specific, while IN vaccines generate a robust cell-mediated response and mucosal antibodies, which are less strain specific. However, IN route may not be a desirable or practical route to vaccinate large numbers of sows or adult pigs. LAIVs are the only vaccines that generate mucosal immunity and are a promising tool in the prevention of IAV-S, but they can replicate in the host and reassortment with field strains cannot be ignored. In fact, reassortment capabilities of LAIVs were demonstrated in experimental conditions and in the field (115, 130). Although this reassortment did not result in increased virulence, further research will be needed to evaluate the impact of this process on influenza A virus's epidemiology. All vaccines mentioned were tested in controlled laboratory environments, which greatly differ from those encountered in the field. Most pigs in the field have already a pre-existing immunity at the time of vaccination, either from MDAs or due to previous infections. Some studies demonstrated that MDAs reduced the efficacy of inactivated and, to a lesser extent, LAIV vaccines. However, other experimental vaccines, such as viral vector vaccines, have never been tested in the presence of MDAs or active immunity. Differences in vaccination rates and region-specific perceptions may also impact vaccine research and commercial products available. For example, while VAERD has been broadly described using commercial and experimental inactivated vaccines in the USA, it has never been described in Europe. This together with the fact that vaccine uptake is much lower in Europe and that regulatory requirements are stricter to update current vaccines when compared with the US, may explain the presence of very old vaccines in the European market. In addition, this context may also explain why United States researchers have actively evaluated innovative vaccine technologies such as LAIVs whereas in Europe efforts focused primarily on the optimization of inactivated vaccines. In China, the availability of only monovalent or bivalent vaccines contrasts the much more complex epidemiological situation in that region.

Although considerable work has been performed to create novel vaccines, investigate their value, and evaluate alternative platforms to control the spread of IAV-S, much more research needs to be done. It is the responsibility of researchers throughout the world to continue working together, not only on improved vaccination strategies, but also to significantly booster worldwide surveillance in an effort to maintain the clearest possible picture of IAV-S epidemiology.

## Author Contributions

JM drafted the first manuscript. JM, DP, AM, and MB wrote the paper. All authors contributed to the article and approved the submitted version.

## Conflict of Interest

The authors were employed by the company Zoetis.
